# Telemedicine in the National Immunization Program (Brazil): A promising tool

**DOI:** 10.1016/j.jvacx.2022.100188

**Published:** 2022-06-27

**Authors:** Luciana Gomes Pedro Brandão, Marcellus Dias da Costa, Pedro Silva Martins, Sergio Carlos Assis de Jesus-Junior, Daniele Fernandes de Aguiar, Alberto Santos de Lemos, Diogo Vicente Bittencourt Sacramento Dias, Charbell Miguel Haddad Kury, Lauro Amaral de Oliveira, Valter Montes de Almeida, Flavio de Carvalho, Ananza Tainá da Silva Santos, José Cerbino-Neto, Margareth Catoia Varela

**Affiliations:** a Centro de Referência para Imunobiológicos Especiais, Instituto Nacional de Infectologia Evandro Chagas (INI – Fiocruz), Rio de Janeiro, Brasil; b Laboratório de Pesquisa em Imunização e Vigilância em Saúde (LIVS), Instituto Nacional de Infectologia Evandro Chagas (INI – Fiocruz), Rio de Janeiro, Brasil; c Serviço de Tecnologia da Informação e Comunicação, Instituto Nacional de Infectologia Evandro Chagas (INI – Fiocruz), Rio de Janeiro, Brasil; d Centro de Referência para Imunobiológicos Especiais, Regional Norte Fluminense – Secretaria Municipal de Saúde de Campos dos Goytacazes, Rio de Janeiro, Brasil; e Centro de Referência para Imunobiológicos Especiais, Itaperuna, Rio de Janeiro, Brazil; f Secretaria Estadual de Saúde do Rio de Janeiro, Gerência de Imunização, Rio de Janeiro, Brasil; g D’Or Institute for Research and Education (IDOR), Rio de Janeiro, Brazil

**Keywords:** Telemedicine, Vaccines, Special immunization, Brazilian National Immunization Program

## Abstract

The coronavirus disease 2019 pandemic abruptly changed the dynamics of basic health care, with the consequent need for adjustments in essential services. The objective of this study was to evaluate the acceptance and impact of telemedicine at a Reference Center for Special Immunobiologicals (CRIE).

**Methods:**

Patients aged 18 years or older who had a medical referral to CRIE and agreed to have a telemedicine consultation were included. After the medical appointments, participants answered a satisfaction survey.

**Results:**

From April 2021 to February 2022, 702 telemedicine consultation were conducted. Over 3,380 vaccines were prescribed via telemedicine. Of all the participants who answered the satisfaction questionnaire, 99.8% stated that they would recommend the service to other people.

**Conclusions:**

Telemedicine proved to be promising tool for healthcare at CRIE and had good acceptance by users, potentially improving access and extending the reach of the National Immunization Program.

## Introduction

Brazil has a outstanding public immunization program. Starting in 1973, the National Immunization Program (PNI) of the Ministry of Health (MoH) currently has more than 38,000 vaccine rooms throughout the national territory [1] and administers 16 different types of vaccines for children, 6 for adolescents, 5 for adults and 3 the older adults, free of charge.

Besides the vaccines recommended in the basic immunization schedule, the PNI offers additional vaccines for patients with chronic diseases or individuals who need to receive differentiated immunobiologicals through the Reference Centers for Special Immunobiologicals (CRIEs). Gradually implemented since 1993, the country currently has 52 CRIE units, at least one in each state of the federation [1]. The distribution of the CRIEs in numbers per region and the resources available in each unit are very heterogeneous. In the standard practice for consultation with the CRIE, the patient has to go personally to the unit with a written medical referral indicating the clinical condition for eligibility to have the first-evaluation. As demand is spontaneous with no appointment, the waiting time can be long and, in some units, it may exceed the service capacity for the day and the patient has to return another day.

Rio de Janeiro has four CRIE units: two in the capital, one in the city of Campos dos Goytacazes, and one in Itaperuna city. In CRIEs, special immunobiologicals are provided (as per the guidelines of MoH) to a patient only after his evaluation by a CRIE professional (physician or nurse) in a face-to-face assessment [2].

The centralization of care in CRIEs allows the rational use of special immunobiologicals that generally have a high cost for the PNI, ensuring that they are used in individuals with greater potential benefit. However, the centralization turns the access more difficult to individuals living outside the municipalities that host CRIEs. Increasing the reach of CRIEs should be a priority for the PNI. Nevertheless, the cost of investment in infrastructure and lack of availability of skilled professionals are factors that hinder the expansion of CRIEs in the country. Moreover, since it is a continent-sized country, an increase in the number of CRIE units may not be sufficient to ensure widespread access to special immunobiologicals. The coronavirus disease 2019 (COVID-19) pandemic caused by the severe acute respiratory syndrome virus 2 (SARS-CoV-2) resulted in the decline of regular vaccination coverage in several regions of the world, including Brazil, and has led to the imminent need for new strategies to ensure access to vaccination [3], [4], [5], [6]. The objective of this study was to evaluate the applicability of telemedicine at a CRIE unit in the context of a pandemic.

## Materials and methods

The study was conducted at the CRIE of Evandro Chagas National Institute of Infectious Diseases (INI/Fiocruz) from April 2021 to February 2022. Patients aged 18 years or older were eligible for virtual consultation. The participants signed an electronic informed consent form (eICF) after reading it. Then they filled in the online consultation request form and uploaded the referral letter to CRIE. After a study team member assessed their eligibility, confirmation of the appointment was sent by e-mail with instructions and the link to access the virtual consultation.

The consultation between the physician and the patient took place in a virtual private room through the Brazilian University Telemedicine Network (RUTE) and lasted 20 min. Study data were collected and managed using REDCap electronic data capture tools hosted at INI/Fiocruz [7]. The collected data were the same as in the face-to-face consultation and inlcuded: gender, age, birthplace, home address, education level, professional activity, clinical condition for referral to CRIE, requested vaccines, comorbidities, use of immunosuppressive drugs, allergies to vaccine or vaccines components, history of needle phobia, previous surgeries, history of vaccine-preventable diseases and previous immunizations. After the end of the consultation, the patient received an e-mail with the attachments of the electronically signed documents (vaccine prescription, and if necessary, laboratory test request, and medical statements) and a link to fill out a satisfaction survey.

During the study period, the standard service at CRIE of INI/Fiocruz remained in normal operation, with first-time face-to-face consultation by spontaneous demand; telemedicine has been implemented as an aditonal and alternative way of medical appointment.

This study was approved by the Institutional Review Board of INI-Fiocruz (CAAE 31647920.4.0000.5262).

## Results

From April 2021 to February 2022, 1176 participants signed the eICF, 820 individuals were eligible for one or more consultations, 907 virtual consultations were scheduled, 702 consultations were conducted (for 687 unique patients: 674 patients had 1 consultation, 12 had 2 consultations and 1 had 4 consultations) and 205 were not conducted because the patients did not connect to the telemedicine platform on the scheduled time. The most frequent reasons for ineligibility were incomplete form or documents.

The mean time between participant registration and telemedicine medical care was 6.69 days. The type of care provided was classified according to demand into: vaccination schedule (n = 635), questions about vaccines (n = 37), assessment of adverse event following vaccination (n = 27), and information exchange between health professionals (n = 3). Among the attended participants, 350 (49.9%) were referred from public institutions and 352 (49.9%) from private ones and 40.7% needed help to schedule the online consultation.

The basic characteristics of the study population are represented in Table 1.

**Table 1:** Characteristics of the study population (n 687 unique patients).

**Variables**	**No (%)**
Gender Female	420 (61.1%)
Age (mean)	52.7
Age group	
18–44	241 (35.1%)
45–64	253 (36.8%)
65–75	117 (17%)
>75	76 (11.1%)
Level of education	
No schooling	21 (3%)
Elementary School	184 (26.2%)
Completed high school	271 (38.6%)
Completed university education	211 (30.1%)
Primary clinical condition (most relevant)*	
Nephropathy	137
Pneumopathy	115
Autoimmune disease	80
Candidates for solid organ transplantation	73
Therapeutic immunosuppression	69
Cardiopathy	67
HIV/AIDS	58
Diabetes mellitus	54
Hematological neoplasm	40
Solid tumor	24
Inflammatory bowel disease	21
Post solid organ transplantation	21
Neurological disease	20
Splenectomy	13
Post bone marrow transplant	12
Hepatopathy	8
Immunodeficient household contact	4
Cerebrospinal fluid leak	3
Distance in km from home address to CRIE**	
250–1827 km	7
100–249 km	30
50–99 km	15
30–49 km	109
16–29.9 km	84

* Patients could have more than one condition.

** Distance calculated by google map between patient’s address and CRIE at Fiocruz for participants living outside Rio de Janeiro city.

Of the total number of unique participants, 442 (64.4%) lived in Rio de Janeiro city, 238 (34.6%) lived outside the municipality of Rio de Janeiro in 35 different cities, and 7 (1%) lived in other states (three in São Paulo, one each in the Federal District, Bahia, Minas Gerais, Santa Catarina). For participants living outside Rio de Janeiro city, 42 (17.1%) informed by telephone call that they managed to have all vaccines in the basic unit next to home, 35 (14.3%) had some vaccines next to home and others at CRIE/INI, 127 (51,8%) had all vaccines at CRIE/INI, 1 vaccinated at private clinic, 1 vaccinated at CRIE in another State, 16 (6.5%) have not been vaccinated at the time of the telephone call. For 12 participants vaccines were not prescribed, 3 patients died and 8 participants didn’t answer the telephone call.

**Fig. 1:**
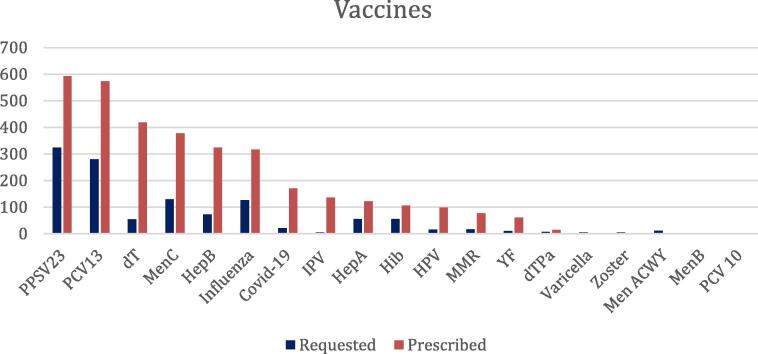
Vaccines requested and prescribed by telemedicine in the study period *PPSV23*: pneumococcal polysaccharide vaccine 23; PCV13: pneumococcal conjugate vaccines 13; dT: diphtheria and tetanus; MenC: meningococcal C; HepB: hepatites B; IPV: inactivated polio vaccine, HepA: hepatites A; Hib: *Haemophilus influenzae* type b; HPV: human papillomavirus; MMR: measles, mumps and rubrella; YF: yellow fever; dTPa: diphtheria, tetanus, and acelular pertussis; MenACWY: meningococcal ACWY, MenB: meningococcal B, PCV10: pneumococcal conjugate vaccines 10. (For interpretation of the references to colour in this figure legend, the reader is referred to the web version of this article.)

Over 3,388 vaccines were prescribed via telemedicine, and the most frequent vaccines prescribed were: 23-valent pneumococcal (n = 593), 13-valent pneumococcal (n = 573), dT (diphtheria and tetanus) (n = 419), and meningococcal C (n = 377) vaccines **(**Fig. 1**).**

The vaccines prescribed by the CRIE team were in accordance with the vaccines requested in the medical referral in only 55 (8.8%) of the cases, in 175 (27.9%) no specific vaccines were indicated in the referral and in 397 (63.3%) there was modification of the requested vaccines, most often (in 96%) by adding unsolicited vaccines**.**

Of the total number (n = 546) of participants who completed the satisfaction questionnaire, 96% reported that the telemedicine consultation met their expectations, 99.3% considered that the experience with the video consultation was good or very good, and 99.8% said that they would recommend the service to friends and relatives. Only one person considered telemedicine a bad experience and would not recommend the service to others (female, 65 years-old). The other participant (female, 56 years-old) that ranked the experience with video consultation as bad, did not manage to turn the video on, but would recommend the service to others.

## Discussion

Telemedicine is a significant advancement in health care delivery in the 21st century, and was boosted by COVID-19 pandemic in various part of the world [8], [9], [10]. Before the COVID-19 pandemic, telemedicine had a minimal role in Brazil, with regulations only for exchange of information between health professionals and for second opinions from specialists. With the pandemic, care via telemedicine had to be expanded in the country to ensure the population's access to basic health care. The MoH published Ordinance 467 on March 20, 2020, temporarily regulating telemedicine in Brazil, including “pre-clinical care, care support, consultation, monitoring and diagnosis through information and communication technology within the scope of the National Health System (SUS), as well as in supplementary and private health” [11]. Although there was no previous experience with care via telemedicine in Brazil, the degree of user acceptance in this study was high and more than 99% of participants were highly satisfied and reported that they would recommend the service to friends and relatives.

The CRIE of INI/Fiocruz was the first in the country to provide care via telemedicine, using an information technology structure already available in the Rute Network and a secure database system of free and open access (REDCap). With the telemedicine tool, CRIE/INI was able to expand its geographic coverage and the monthly number of consultations. During the study period, 3184 face-to-face first-time adult consultations were taken at CRIE, and telemedicine represented 18.1% of the total number of consultations in the first year of implementation.

In this study, telemedicine consultation resulted in 3,388 prescribed vaccines, and in 91,2% of the consultations, the medical evaluation at CRIE had an important impact on the vaccination schedule, in 175 (27.9%) no specific vaccines were indicated in the referral and in 397 (63.3%) a modification of the requested vaccine had to be done. The dT was the vaccine most frequently added to the prescription, showing the lack of adult’s vaccination culture in Brazil and the absence of medical referral to this vaccine. dT has been part of the basic vaccination schedule for all ages for decades and is widely available in basic health units in Brazil. Although the number of actually administered vaccines as a result of telemedicine consultation could not be directly measured in this study, the fact of having a written vaccine prescription after a medical evaluation turns out possible to the patient to have some vaccines administered in basic units next home and others in CRIE units or private clinics.

The results of this study show that the telemedicine service at CRIE had a high degree of satisfaction and served adults of various age groups (up to 27.5% had 65 years or more), with different levels of education (69% had up to high school education), and with different underlying diseases. The description of primary medical condition of the studied population reflects the stand of care of specialist physicians for referral to special immunizations. For some baseline conditions, referral to CRIE is already an established routine. However, for other situations, such as diabetes mellitus and solid tumors, the number of referrals is still very small for the number of patients affected. This can help to guide the National Immunization Program to launch campaigns and training in immunization for specialists in other areas.

About 35% of the participants lived outside the city of Rio de Janeiro, and 31.4% of these participants had all or some vaccines administered at basic units next to home, showing the potential of telemedicine to expand access to special vaccines in the national territory, especially if there is a partnership with the state to send the prescribed vaccines to the nearest vaccine units. As the distribution of basic vaccines is made in a regular basis from the state distribution center to the municipalities, it is feasible to send special vaccines by demand.

The main limitation of this study was the considerable percentage of non-attendance in virtual consultations – it was approximately 22,6% (205/907) of the scheduled visits. This can be explained, in part, due to the lack of previous experience of the study participants with online medical care and low literacy level. Another limitation is the lack of comparators with the face-to-face consultation in CRIE-INI, which was not foreseen in this study, but can be easily done in the future.

The use of eHealth tools and technology in immunization is positive and promising [12]. This pilot study demonstrated that telemedicine is a promising tool to assist patients at a CRIE in a developing country with no established history of online consultations before the SARS-CoV-2 pandemic. It increased the population's access to expert immunization advice and facilitated access to special immunobiologicals. In the era of anti-vaccine movements, access to ftrustworthy information about vaccines can help maintain public confidence in vaccines and contribute to high vaccination coverage. This project represents an innovation in the immunization program in Brazil and highlights its potential for expansion at the national level.

## Funding

The project was supported by independent quality improvement grant from Pfizer Global Medical Grants [grant number ID#63732937]. The funder had no role in study design; in the collection, analysis and interpretation of data; in the writing of the report; and in the decision to submit the article for publication.

## CRediT authorship contribution statement

**Luciana Gomes Pedro Brandão:** Conceptualization, Methodology, Software, Formal analysis, Investigation, Data curation, Writing – original draft, Visualization, Funding acquisition. **Marcellus Dias da Costa:** Methodology, Software, Investigation, Data curation, Writing – review & editing. **Pedro da Silva Martins:** Methodology, Software, Investigation, Data curation, Writing – review & editing. **Sergio Carlos Assis de Jesus Junior:** Data curation, Writing – review & editing. **Daniele Fernandes de Aguiar:** Data curation, Writing – review & editing. **Alberto dos Santos Lemos:** Methodology, Software, Writing – review & editing. **Diego Vicente Bittencourt Sacramento Dias:** Software, Data curation, Writing – review & editing, Supervision. **Charbell Miguel Haddad Kury:** Methodology, Writing – review & editing. **Lauro Amaral de Oliveira:** Methodology, Writing – review & editing. **Valter Montes de Almeida:** Methodology, Writing – review & editing. **Flávio de Carvalho:** Methodology, Writing – review & editing. **Ananza Tainá da Silva Santos:** Methodology, Writing – review & editing. **José Cerbino-Neto:** Methodology, Writing – review & editing, Supervision. **Margareth Catoia Varela:** Conceptualization, Methodology, Software, Formal analysis, Data curation, Writing – original draft, Visualization, Supervision.

## Declaration of Competing Interest

The authors declare the following financial interests/personal relationships which may be considered as potential competing interests: ‘Brandao, LGP reports financial support was provided by Pfizer Inc.’
